# Breast Cancer-Associated Venous Thromboembolism: Risk Factors, Mechanisms, and Clinical Management

**DOI:** 10.3390/cancers18091486

**Published:** 2026-05-05

**Authors:** Panlin Xie, Yunbo Luo, Lingmi Hou, Jia Xu, Qun Yi

**Affiliations:** 1 Department of General Internal Medicine, Sichuan Clinical Research Center for Cancer, Sichuan Cancer Hospital & Institute, Sichuan Cancer Center, School of Medicine, University of Electronic Science and Technology of China, Chengdu 610000, China; 202521130226@std.uestc.edu.cn; 2 Department of Breast Surgery, Sichuan Clinical Research Center for Cancer, Sichuan Cancer Hospital & Institute, Sichuan Cancer Center, University of Electronic Science and Technology of China, Chengdu 610000, China; 3 Department of Plastic Surgery, Sichuan Clinical Research Center for Cancer, Sichuan Cancer Hospital & Institute, Sichuan Cancer Center, University of Electronic Science and Technology of China, Chengdu 610000, China; 4 Department of Medical Oncology, Sichuan Clinical Research Center for Cancer, Sichuan Cancer Hospital & Institute, Sichuan Cancer Center, University of Electronic Science and Technology of China, Chengdu 610000, China

**Keywords:** breast cancer, venous thromboembolism, risk factors, tumor metastasis, clinical management

## Abstract

Venous thromboembolism (VTE) is a clinically significant complication in patients with breast cancer (BC) and may disrupt treatment continuity while contributing to adverse outcomes. Although BC is generally regarded as a relatively low-risk malignancy for VTE compared with several other cancer types, its high incidence and the increasing use of multimodal therapies have resulted in a growing clinical burden of breast cancer-associated VTE. This review summarizes the epidemiological features, risk factors, biological mechanisms, and advances in the prevention and management of breast cancer-associated VTE. Current evidence indicates that patients with cancer have an approximately 4- to 7-fold higher risk of VTE than the general population, with the risk in BC being particularly pronounced during the first 3–6 months after diagnosis. Older age, metabolic comorbidities, advanced disease, and exposure to multiple anticancer therapies have all been associated with an increased risk of VTE. Mechanistically, tissue factor, procoagulant extracellular vesicles, neutrophil extracellular traps, and inflammatory signaling pathways may contribute to breast cancer-associated VTE by promoting coagulation activation and endothelial dysfunction, while also linking thrombosis to immune evasion and Smetastatic progression. Improved identification of high-risk patients, optimization of dynamic risk assessment, and the implementation of individualized prophylactic and anticoagulant strategies may help improve outcomes in patients with BC.

## Simple Summary

Venous thromboembolism (VTE) is a serious complication in patients with breast cancer and is influenced by both tumor-related and treatment-related factors. Increasing evidence suggests that breast cancer-associated VTE arises from complex interactions among tumor biology, coagulation, inflammation, and the tumor microenvironment. These processes may not only promote thrombosis but also be linked to tumor progression and metastasis. This review summarizes current evidence on the epidemiology, mechanisms, risk factors, and clinical management of breast cancer-associated VTE. It also highlights the limitations of current biomarkers and risk assessment tools, as well as the need for more breast cancer-specific evidence to support better risk stratification, prevention, and individualized care.

## 1. Introduction

BC is one of the most common malignancies in women worldwide and remains a leading cause of cancer-related death. A recent 2025 analysis based on the GLOBOCAN 2022 database, covering 185 countries and territories, estimated that there were approximately 2.3 million new cases of BC and 670,000 deaths worldwide in 2022. By 2050, these numbers are projected to rise to 3.2 million and 1.1 million, respectively, indicating a continuing increase in the global burden of BC [1]. As survival among patients with BC has improved substantially, the adverse impact of associated complications, particularly venous thromboembolism (VTE), on clinical outcomes has become increasingly apparent. VTE is a common and potentially fatal complication in patients with cancer and may lead to treatment interruption, reduced quality of life, and poor prognosis [2]. It also has a bidirectional relationship with tumor metastasis and disease progression. In recent years, professional societies including ASCO, ESC, and ISTH have updated guidance on cancer-associated VTE, further underscoring the importance of thrombotic risk assessment, prevention, and standardized management in patients with cancer [3,4,5]. Accordingly, this review aims to provide a systematic overview of the epidemiology, risk factors, underlying mechanisms, and advances in the clinical management of breast cancer-associated VTE. In addition to summarizing the clinical features and current management of this condition, it highlights the bidirectional relationship between BC and thrombosis. Particular attention is given to the roles of procoagulant factors, immune–inflammatory responses, and the tumor microenvironment in thrombosis and tumor progression. By integrating mechanistic insights with clinical management from a breast cancer-specific perspective, this review aims to inform risk stratification and guide more precise preventive and therapeutic strategies.

## 2. Epidemiology and Prognosis of VTE in Breast Cancer

### 2.1. VTE Risk in Patients with and Without Cancer and the Relative Risk Profile of Breast Cancer

Malignancy is a major risk factor for VTE. Large population-based studies have shown that patients with cancer have an approximately 4- to 7-fold higher risk of VTE than individuals without cancer [6]. More recent population-based cohort data further indicate that the 12-month cumulative incidence of VTE is approximately 2.3% in patients with cancer, compared with 0.35% in non-cancer controls [7]. These findings indicate that cancer increases not only the relative risk of VTE but also the overall burden of thrombotic events.

VTE risk varies substantially across different malignancies. Compared with high-risk cancers such as pancreatic cancer, brain cancer, and lung cancer, BC is generally classified as a relatively low-risk malignancy for VTE. In large population-based analyses, the relative risk of VTE in BC has been estimated at approximately 3.1–3.3, which is clearly lower than that observed in several high-risk tumor types [6]. However, this relatively lower risk does not diminish its clinical relevance. Given the high prevalence of BC and the additional thrombotic risk associated with multimodal treatment, the harm of breast cancer-associated VTE remains severe.

### 2.2. Absolute Risk and Time-Dependent Patterns of Breast Cancer-Associated VTE

Absolute event rates and time-dependent patterns provide a more informative assessment of the true clinical burden of breast cancer-associated VTE than relative risk estimates alone. Cohort studies have shown that the risk of VTE among patients with BC is time-dependent and varies across disease stages and treatment exposures. Walker et al. reported that VTE risk is highest during chemotherapy and in the first month after treatment, with an annualized incidence of approximately 6%. During tamoxifen treatment, the annualized incidence is approximately 2%, representing an approximately 4-fold increase compared with the pretreatment period. These findings suggest that systemic treatment exposure is an important determinant of VTE risk in patients with BC [8].

More recent cohort studies, particularly those published after 2020, have further refined the absolute risk profile of breast cancer-associated VTE. In a contemporary population-based cohort study, Mulder et al. reported that the 12-month cumulative incidence of VTE was approximately 2.3% in patients with cancer, compared with 0.35% in non-cancer controls, indicating that cancer increases not only the relative risk of VTE but also the absolute burden of thrombotic events [7]. Concerning breast cancer-specific evidence, a subgroup analysis from the Cancer-VTE Registry showed a baseline VTE prevalence of 2.0% at screening before treatment initiation and a composite VTE incidence of 0.5% during approximately 1 year of follow-up, providing important recent data on the absolute risk of VTE in BC [9]. In addition, postoperative follow-up data suggest that the risk of breast cancer-associated VTE may accumulate over time, with a higher risk observed particularly in patients undergoing immediate reconstruction, especially autologous reconstruction [10].

Overall, although BC is not among the highest-risk malignancies for VTE, its absolute thrombotic risk remains clinically relevant during the early period after diagnosis, systemic treatment exposure, the perioperative setting, and disease progression. Key studies and their main findings are summarized in Table 1.

This table summarizes representative data on the absolute risk and time-dependent patterns of breast cancer-associated VTE. The corresponding studies are listed in references [7,8,9,10].

### 2.3. Prognostic Significance and Competing Risk

VTE is not only an important complication in patients with BC, but it is also associated with poor prognosis. In a population-based cohort of patients with BC, VTE remained significantly associated with an increased risk of death after adjustment for confounders and modeling VTE as a time-dependent exposure, with an adjusted hazard ratio of 2.42 (95% CI 2.13–2.75) [11]. This finding suggests that VTE is independently associated with an increased risk of death in patients with BC.

It should be noted that death is an important competing event in studies of breast cancer-associated VTE, particularly in patients with advanced disease. In the BC subgroup of the Cancer-VTE Registry, the composite VTE incidence was 0.5% over approximately 1 year of follow-up, while the all-cause mortality rate was 2.1% [9]. This indicates that, in studies of breast cancer-associated VTE, death should not be treated simply as a censoring event. If death is not treated as a competing risk, conventional survival analyses may overestimate the cumulative incidence of VTE. Therefore, in epidemiological studies of breast cancer-associated VTE, absolute risk estimates derived from cumulative incidence functions or Fine–Gray models should be reported whenever possible, in addition to relative risk estimates, to improve clinical interpretability.

## 3. Risk Factors for VTE in BC Patients

**Figure 1:**
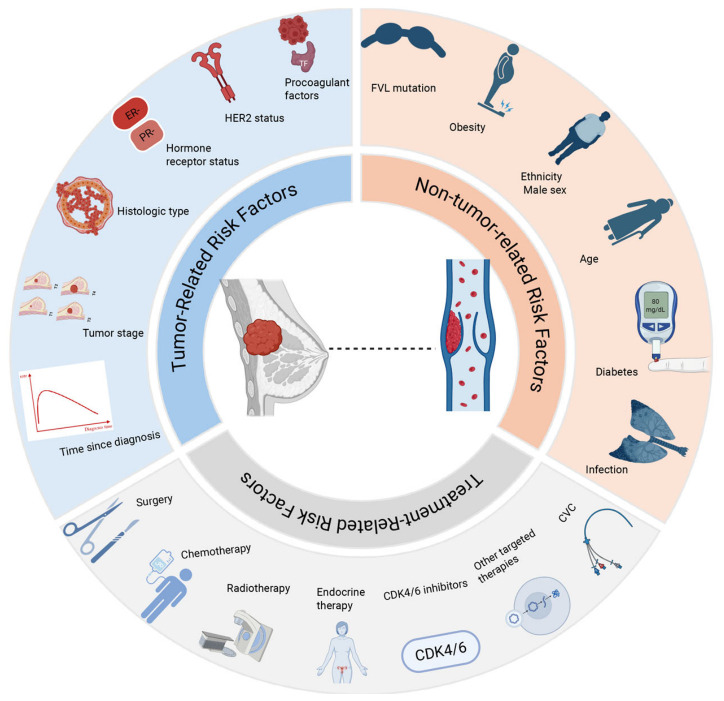
Risk factors for VTE in BC. This figure was designed by the authors and created with BioRender.com. Abbreviations: BC, breast cancer; VTE, venous thromboembolism; ER, estrogen receptor; PR, progesterone receptor; HER2, human epidermal growth factor receptor 2; FVL, factor V Leiden; TF, tissue factor; CVC, central venous catheter; CDK4/6, cyclin-dependent kinase 4/6.

The risk factors for VTE in BC patients are multifaceted and can be categorized into three primary domains (Figure 1). Firstly, patient-related factors include advanced age, obesity, and inherited thrombophilia. Secondly, tumor-specific characteristics encompass advanced disease stage, pathological subtypes, and specific molecular profiles, such as HER2-positive status. Most critically, anticancer therapies contribute to a cumulative risk. Modalities such as chemotherapy, central venous catheterization, extensive surgery (e.g., axillary lymph node dissection), and the use of specific anti-angiogenic targeted agents and hormonal therapies often interact synergistically. All these factors elevate the risk of thrombosis longitudinally across the entire spectrum —from diagnosis through treatment to subsequent follow-up.

### 3.1. Non-Tumor-Related Risk Factors for VTE in Breast Cancer

#### 3.1.1. Genetic Factors

VTE is closely associated with inherited thrombophilia. Current evidence indicates that Factor V Leiden (FVL), a mutation in the *F5* gene, is one of the more clearly established hereditary thrombophilic factors. In a prospective study, cancer patients carrying the FVL mutation had a higher risk of VTE than non-carriers, with a hazard ratio of 2.0 (95% CI 1.0–4.0) [12]. In addition to FVL, other classical inherited thrombophilic factors, including the prothrombin G20210A mutation [13] and deficiencies of protein C and protein S [14], may also increase thrombotic susceptibility in patients with cancer. In recent years, the potential value of polygenic risk scores (PRS) in risk stratification for cancer-associated VTE has gained increasing attention. This suggests that genetic predisposition may help identify high-risk individuals beyond conventional clinical risk factors [15]. Taken together, these findings suggest that inherited thrombophilia may have value for risk stratification for VTE risk assessment in patients with BC.

#### 3.1.2. Patient Characteristics

Patient-related factors such as age, sex, race, and obesity are associated with VTE risk in patients with BC. Available evidence suggests that older age is associated with an increased risk of VTE in this population. A cohort study based on the SEER-Medicare database showed that patients aged 80 years or older had an approximately 5-fold higher risk of VTE than younger patients [16]. Although male BC is rare, available evidence suggests that men with BC may have a higher risk of VTE than women [16]. Racial differences have also been reported, with African American patients having a higher risk of VTE, whereas Asian/Pacific Islander patients appear to have a relatively lower risk in some population-based cohorts [17]. Obesity is also an important risk factor. Current evidence indicates that patients with severe obesity (BMI ≥ 40 kg/m^2^) have a significantly increased risk of VTE compared with those with normal body weight (HR = 3.0, 95% CI 2.1–4.4) [8]. Taken together, these patient-related characteristics reflect important host susceptibility factors in BC and may help inform clinical risk stratification.

#### 3.1.3. Comorbidities Such as Diabetes and Infection

Diabetes, pulmonary disease, renal disease, anemia, infection, and other comorbidities may increase thrombotic susceptibility in patients with cancer and have also been considered in breast cancer-specific comorbidity analyses [18,19]. These conditions may reflect a higher baseline clinical burden and may contribute to VTE risk through overlapping mechanisms, including inflammation, vascular dysfunction, and reduced mobility. In breast cancer cohorts, comorbidity burden and individual conditions such as pulmonary disease, renal disease, infection, anemia, and prior arterial thromboembolism have been associated with increased thromboembolic risk [16,19]. Therefore, VTE risk assessment in patients with BC should not focus solely on tumor-related factors but should also take into account the patient’s baseline clinical status and comorbidity burden.

### 3.2. Tumor- and Treatment-Related Risk Factors

#### 3.2.1. Tumor-Related Risk Factors

Current evidence indicates that the risk of breast cancer-associated VTE shows a clear time-dependent pattern, with the first 3–6 months after diagnosis representing a high-risk period. Population-based cohort data show that the risk of VTE is highest within the first 6 months after BC diagnosis (HR = 8.62, 95% CI 6.56–11.33) [20]. Tumor-related characteristics such as pathological type, tumor stage, and molecular subtype also influence VTE risk. Compared with patients with non-invasive lesions such as ductal carcinoma in situ (DCIS) and lobular carcinoma in situ (LCIS), patients with invasive BC have a higher risk of VTE [10]. The risk also increases with advancing stage, with the greatest risk observed in patients with distant metastases. In a cohort study including 89,841 patients with BC, the risk of VTE increased by 22%, 39%, and 98% in stage II, III, and IV disease, respectively, compared with stage I disease [16]. Differences have also been observed among molecular subtypes, with triple-negative and HER2-positive BC generally associated with higher VTE risk, whereas luminal subtypes tend to have a lower baseline risk [21,22]. Taken together, tumor burden, invasiveness, and biological characteristics are important tumor-related determinants of VTE risk in patients with BC.

#### 3.2.2. Treatment-Related Risk Factors

Treatment-related VTE risk in BC is primarily associated with surgery, chemotherapy, central venous catheterization, endocrine therapy, CDK4/6 inhibitors, and other systemic treatments. Overall, the risk of surgery-related VTE is relatively low, although it may rise transiently during the early postoperative period [23]. A UK cohort study showed that the risk of VTE was increased during the first month after BC surgery (HR = 2.2, 95% CI 1.4–3.4) [8]. Chemotherapy is a relatively well-established prothrombotic exposure. Population-based evidence suggests that chemotherapy is associated with an increased risk of VTE in patients with BC, and this risk may be further modified by genetic susceptibility [24]. Central venous catheterization is also a well-recognized clinical risk factor. A meta-analysis reported that the overall incidence of peripherally inserted central catheter-related thrombosis in patients with BC was 7.0% (95% CI 4.0–13.0%), increasing to 12.9% (95% CI 7.0–22.5%) after adjustment [25]. Among endocrine therapies, tamoxifen has been consistently associated with an increased risk of VTE; a Danish population-based cohort study reported a higher risk with tamoxifen use (RR = 3.5, 95% CI 2.1–6.0) [26]. By contrast, aromatase inhibitors appear to confer a lower VTE risk than tamoxifen [27,28]. However, risk estimates across studies are not entirely uniform and may be influenced by differences in menopausal status, treatment duration, follow-up window, and adjustment for confounding factors (Table 2). In recent years, the thrombotic risk associated with CDK4/6 inhibitors has received increasing attention, although potential differences among individual agents remain uncertain, and the available evidence is not entirely consistent [29,30]. Evidence regarding other targeted therapies also remains limited. The contribution of radiotherapy to VTE risk in BC remains uncertain; however, available evidence suggests that it may add to the overall thrombotic burden in selected high-risk patients [31,32]. Overall, treatment exposure represents one of the most clinically modifiable components of VTE risk in BC. Accordingly, VTE risk should be dynamically assessed and reassessed according to treatment stage, concomitant therapies, catheter status, and baseline thrombotic risk.

## 4. Impact of Breast Cancer-Associated Venous Thromboembolism on Patients

### 4.1. Survival and Prognostic Significance

VTE is not only a major complication in patients with BC but is also associated with poorer survival [33]. Previous cohort studies in patients with BC have shown that, even when VTE was treated as a time-dependent exposure in adjusted analyses, the development of VTE remained significantly associated with an increased risk of death, with an adjusted HR of 2.42 (95% CI 2.13–2.75) [11]. This suggests that the adverse prognostic impact of VTE is unlikely to be explained solely by baseline risk differences and may also reflect disease progression and deterioration in overall clinical status. However, survival estimates may vary across studies depending on how VTE is defined, whether it is modeled as a time-dependent exposure, and whether important factors such as age, stage, and treatment are adequately adjusted for. Therefore, adjusted survival analyses should be prioritized when evaluating the prognostic significance of breast cancer-associated VTE. Overall, VTE in patients with BC is not only an important complication during the disease course but also a clinically important marker of poor prognosis.

### 4.2. Impact on Quality of Life and Functional Status

VTE can substantially impair quality of life in patients with cancer and may have lasting adverse effects on functional status. In addition to acute manifestations such as limb swelling, pain, dyspnea, and reduced functional capacity, recurrent VTE and anticoagulation-related bleeding may further increase the burden on patients [34]. Studies of cancer-associated VTE using validated health-related quality-of-life instruments, including the EQ-5D, have shown that VTE and recurrent thrombotic events are associated with lower health utility scores [35]. These findings suggest that thrombotic events affect not only short-term symptom burden but also overall health status and treatment experience over time. However, most quantitative evidence regarding quality of life has been derived from broader populations with cancer-associated VTE, whereas breast cancer-specific data remain limited. Therefore, caution is warranted when extrapolating these findings to patients with BC.

### 4.3. Healthcare Utilization and Economic Burden

VTE in patients with BC is also associated with increased healthcare utilization and economic burden. Previous studies have shown that, compared with cancer patients without VTE, those with VTE have higher hospitalization rates, longer hospital stays, and significantly greater direct medical costs [36]. A real-world study from the United States reported that the adjusted incremental all-cause healthcare cost associated with VTE in patients with cancer was approximately USD 30,538 per patient, highlighting the substantial economic impact of thrombotic complications [37]. Although breast cancer-specific cost data remain limited, the economic impact of VTE is likely to be considerable given the large number of patients with BC and the costs associated with hospitalization, anticoagulation therapy, recurrence monitoring, and management of complications.

## 5. Interactive Mechanisms Between Breast Cancer and VTE

**Figure 2:**
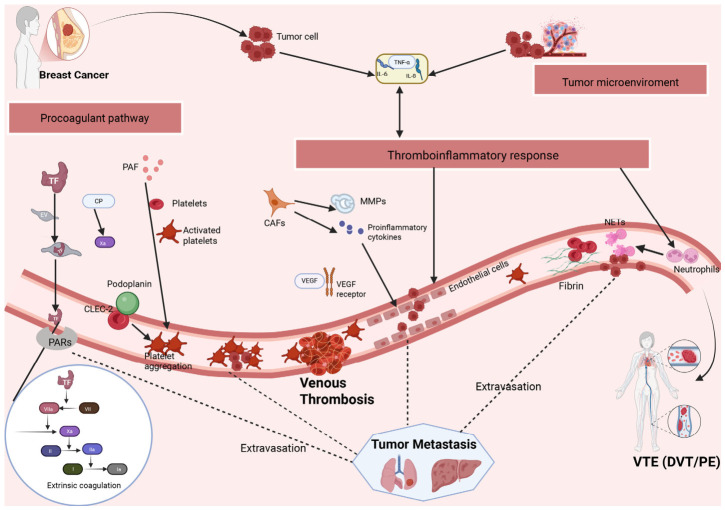
Mechanistic crosstalk between breast cancer, venous thrombosis, and tumor progression. This figure was designed by the authors and created with BioRender.com. In the extrinsic coagulation pathway panel, arrows indicate the activation sequence of coagulation factors; Roman numerals indicate coagulation factors, and “a” denotes the activated form of the corresponding factor. Abbreviations: BC, breast cancer; VTE, venous thromboembolism; TF, tissue factor; EVs, extracellular vesicles; CP, cancer procoagulant; PARs, protease-activated receptors; PAF, platelet-activating factor; CLEC-2, C-type lectin-like receptor 2; CAFs, cancer-associated fibroblasts; MMPs, matrix metalloproteinases; VEGF, vascular endothelial growth factor; NETs, neutrophil extracellular traps; IL-6, interleukin-6; IL-8, interleukin-8; TNF-α, tumor necrosis factor-alpha; FVIIa, activated factor VII; FXa, activated factor X; FII, prothrombin; FIIa, thrombin; FI, fibrinogen; FIa, fibrin; DVT, deep vein thrombosis; PE, pulmonary embolism.

The development of breast cancer-associated VTE is unlikely to be driven by a single factor but rather arises from the interplay of multiple mechanisms, including a tumor-associated procoagulant phenotype, inflammatory and immune responses, endothelial injury, platelet activation, and changes in the tumor microenvironment. BC cells can directly activate the coagulation cascade by expressing procoagulant molecules while also amplifying thrombo-inflammatory responses through interactions with platelets, neutrophils, endothelial cells, and stromal cells. Overall, breast cancer-associated VTE represents a thrombo-inflammatory process initiated by a tumor-associated procoagulant phenotype and progressively amplified by platelet activation, NET formation, and endothelial injury. The major mechanisms underlying breast cancer-associated VTE are summarized schematically in Figure 2. Because evidence strength differs across these pathways, the following discussion distinguishes clinically supported breast cancer-related findings from mechanisms inferred mainly from experimental, preclinical, or broader cancer studies.

### 5.1. Tumor-Associated Procoagulant Phenotype and Activation of the Extrinsic Coagulation Pathway

BC cells can express tissue factor (TF) [38] and release TF-bearing extracellular vesicles (EVs) into the circulation, thereby activating the extrinsic coagulation pathway and promoting thrombin generation and fibrin formation [39,40,41]. In addition to TF, cancer procoagulant (CP) may also contribute to hypercoagulability through direct activation of factor X [42,43]. Recent preclinical studies further suggest that the role of extracellular vesicles in cancer-associated thrombosis extends beyond the transport of procoagulant molecules into the circulation. Small extracellular vesicles (sEVs) released from prothrombotic microenvironments in distant organs may carry clustered integrin β2, which can form a complex with integrin αX. This complex may subsequently interact with platelet surface GPIb, induce platelet aggregation, and thereby promote thrombosis [44]. However, this evidence is currently derived mainly from preclinical studies, and its clinical relevance in BC remains to be established. Some studies have also suggested that TF expression may be more prominent in aggressive BC subtypes, particularly triple-negative BC, possibly reflecting a more pronounced procoagulant and inflammatory tumor phenotype [45,46].

### 5.2. Tumor–Host Cell Interactions in Thromboinflammation

#### 5.2.1. Platelet Activation and Platelet–Tumor Cell Interactions

BC cells may release platelet-activating factors and directly induce platelet aggregation [39,47]. Activated platelets, in turn, release platelet-derived microparticles enriched in procoagulant and angiogenic mediators, thereby reinforcing local coagulation responses and promoting thrombus formation [48]. The podoplanin–CLEC-2 axis has also been implicated in tumor-associated platelet activation. Mechanistic studies suggest that podoplanin expressed by tumor cells or tumor stromal cells can bind to CLEC-2 on the platelet surface, thereby promoting platelet aggregation and potentially contributing to thrombosis [49,50].

#### 5.2.2. NET Formation and Its Prothrombotic Role

In the setting of tumor-associated inflammation, neutrophils can release neutrophil extracellular traps (NETs). NETs, which are composed of DNA, histones, and proteases, can trap platelets and red blood cells, promote fibrin deposition, and facilitate thrombus formation [51]. NETs are therefore not merely products of inflammation but an important link between inflammatory responses to coagulation activation [52].

#### 5.2.3. Endothelial Activation and Procoagulant Phenotypic Transition

Factors released by BC cells and cells within the tumor microenvironment, including VEGF, IL-6, IL-8, and tumor necrosis factor-α, can disrupt endothelial integrity, increase vascular permeability, and induce endothelial cells to express adhesion molecules and procoagulant molecules [41,53,54,55]. Dysfunctional endothelial cells not only promote leukocyte recruitment but also provide a procoagulant surface that favors thrombin generation and fibrin deposition. Importantly, platelet activation, NET formation, and endothelial injury are not isolated events; rather, they constitute an interconnected and mutually amplifying thrombo-inflammatory process that promotes breast cancer-associated VTE [56,57].

### 5.3. Tumor Microenvironment Remodeling and Sustained Hypercoagulability

Breast cancer-associated VTE is also closely linked to changes in the tumor microenvironment. Cancer-associated fibroblasts (CAFs) within the breast tumor stroma can secrete matrix metalloproteinases (MMPs) and proinflammatory cytokines in response to signals from BC cells [58,59]. MMPs promote extracellular matrix degradation and remodeling, thereby reshaping the local microenvironment, while proinflammatory mediators further enhance endothelial activation, platelet activation, and local inflammatory responses [53]. The resulting microenvironment not only supports the persistence of a hypercoagulable state but may also promote local microthrombus formation.

In addition, breast cancer-associated VTE is not solely the result of enhanced coagulation; alterations in fibrinolytic function may also contribute. Some studies suggest that upregulation of fibrinolysis-inhibitory molecules within the tumor microenvironment can reduce fibrin degradation, thereby favoring thrombus formation and persistence [60,61]. Emerging evidence further indicates that elevated levels of fibrinolysis inhibitors such as PAI-1 are associated not only with thrombus stabilization but also with tumor cell migration, angiogenesis, and poor prognosis [62,63,64]. Therefore, the hypercoagulable state associated with BC is better understood as a consequence of the interplay among coagulation activation, inflammatory amplification, and fibrinolytic imbalance.

### 5.4. Non-Tumor Factors in Breast Cancer-Associated Hypercoagulability

In addition to tumor-related mechanisms, non-tumor factors also contribute to the development and persistence of breast cancer-associated hypercoagulability. In obesity, adipose tissue is characterized by chronic low-grade inflammation, and increased secretion of inflammatory cytokines such as IL-6 by adipocytes and infiltrating macrophages may further upregulate TF expression, promote endothelial dysfunction and platelet activation, and thereby create a more prothrombotic milieu [65,66]. Diabetes and insulin resistance may further increase thrombotic risk by promoting coagulation activation, impairing fibrinolysis, worsening vascular dysfunction, and inducing microcirculatory abnormalities [67,68,69]. Meanwhile, age-related vascular changes, a greater burden of comorbidities, and reduced mobility may further reinforce this prothrombotic background in patients with BC [70,71]. Overall, although these non-tumor factors are often recognized clinically as features of high-risk patients, they also contribute to the development and persistence of breast cancer-associated hypercoagulability.

### 5.5. Tumor Burden, Molecular Subtypes, and Treatment-Related Factors

Tumor burden, molecular subtype, and treatment-related factors may further contribute to the development of breast cancer-associated VTE in the context of the mechanisms described above. Advanced-stage disease and greater tumor burden are likely to increase the release of procoagulant factors, and VTE risk is particularly elevated in patients with distant metastases. With respect to molecular subtypes, triple-negative BC and HER2-positive BC may exhibit greater aggressiveness and a more pronounced procoagulant and thrombo-inflammatory phenotype, whereas luminal subtypes tend to have a relatively lower baseline risk [38]. In parallel, treatment exposures such as surgery, chemotherapy, radiotherapy, central venous catheterization, endocrine therapy, and targeted therapy may further promote VTE through endothelial injury, blood flow stasis, the release of procoagulant factors, and the presence of a pre-existing prothrombotic background [29,72,73]. Overall, the biological characteristics of the tumor and treatment exposure are not merely clinically observed risk factors, but they also form an important pathophysiological basis for the development of breast cancer-associated VTE.

### 5.6. Potential Mechanisms Linking Thrombosis to Tumor Metastasis and Disease Progression

Thrombosis is not only a consequence of the hypercoagulable state associated with BC but may also contribute to tumor metastasis and disease progression through several downstream mechanisms. The supporting evidence for these pathways ranges from breast cancer-related studies to broader experimental cancer models.

#### 5.6.1. Platelet-Mediated Survival of Circulating Tumor Cells and Reduced Immune Clearance

Evidence from experimental studies and broader cancer models suggests that activated platelets can adhere to circulating tumor cells and form platelet–tumor cell aggregates, which may shield tumor cells from shear stress and immune-mediated clearance, particularly natural killer cell-mediated cytotoxicity. Breast cancer-related studies further indicate that activated platelets may promote tumor growth, angiogenesis, hematogenous metastasis, and metastatic outgrowth. For example, platelet releasate can enhance breast cancer growth and angiogenesis through VEGF–integrin cooperative signaling [74]. In another breast cancer model, activated platelets were shown to facilitate hematogenous metastasis by modulating the PDGFR-β/COX-2 axis [75]. Recent evidence also suggests that platelets may favor the outgrowth of established metastases [76]. In TNBC, recent evidence further suggests that a procoagulant platelet phenotype may contribute to tumor immune evasion by carrying immune checkpoint molecules and modulating platelet–leukocyte interactions within the tumor microenvironment [77]. Moreover, platelet glycoprotein VI (GPVI) has been reported to promote breast cancer cell metastasis through interaction with tumor cell-derived galectin-3 [78]. Together, these findings support a breast cancer-related role of platelet activation in immune modulation and metastatic progression. However, whether these platelet-mediated mechanisms directly contribute to the occurrence of breast cancer-associated VTE remains to be clarified.

#### 5.6.2. TF/PAR-Related Signaling and Tumor Progression in Aggressive Breast Cancer Subtypes

In BC, TF contributes not only to coagulation activation but also to tumor-related signaling. Some studies suggest that TF-associated complexes may activate protease-activated receptor (PAR)-mediated pathways, thereby promoting tumor cell migration, invasion, and angiogenesis [79]. Thrombin and factor Xa may likewise influence these processes through PAR-dependent signaling in addition to their established roles in thrombosis [80,81]. Available evidence suggests that this mechanism may be of particular relevance in aggressive BC subtypes, especially triple-negative BC. By contrast, although HER2-positive BC is also an aggressive subtype, direct evidence linking HER2-positive disease to TF/PAR-related procoagulant signaling remains relatively limited.

#### 5.6.3. Fibrin Deposition and Microthrombi Formation Facilitate Tumor Cell Adhesion and Extravasation

Fibrin deposition and microthrombi formation may alter the local microcirculatory environment, thereby facilitating the adhesion, retention, and extravasation of circulating tumor cells within distant vascular beds and ultimately promoting metastatic colonization [82,83,84].

#### 5.6.4. NETs and Related Inflammatory Mediators in Premetastatic Niche Formation

NETs and related inflammatory mediators may contribute to premetastatic niche formation by remodeling the extracellular matrix and inflammatory microenvironment and, under certain conditions, may also promote the reactivation of dormant tumor cells [85,86].

#### 5.6.5. Potential Role of Prothrombotic Microenvironment-Derived Extracellular Vesicles in Linking Thrombosis to Metastatic Dissemination

Recent preclinical studies suggest that extracellular vesicles derived from the prothrombotic microenvironment may induce platelet aggregation through an integrin β2-dependent mechanism, thereby promoting thrombosis. Blockade of this pathway has been shown to reduce both thrombus formation and pulmonary metastasis [44]. These findings suggest that the prothrombotic microenvironment and its associated extracellular vesicles may be involved not only in cancer-associated thrombosis but also in mediating the link between thrombosis and metastatic dissemination. It should be noted, however, that the available evidence is derived mainly from preclinical studies, and the clinical relevance of these findings in BC requires further validation.

## 6. Risk Assessment, Prevention, and Treatment Strategies for Breast Cancer-Associated VTE

### 6.1. Risk Prediction and Assessment

Risk assessment for breast cancer-associated VTE should not rely on a single pan-cancer risk scoring model but should instead be integrated into a dynamic, stratified clinical decision-making framework. Current ASH, ASCO, and ESC guidelines all emphasize that decisions regarding thromboprophylaxis and anticoagulant selection should take into account thrombotic risk, bleeding risk, treatment-related exposures, drug–drug interactions, and the patient’s overall clinical condition [3,87,88,89]. The Khorana score may be used for initial screening in ambulatory patients, but it does not adequately capture the risk heterogeneity associated with different BC subtypes and specific treatment exposures [90,91]. The COMPASS-CAT model incorporates more information on treatment and comorbidities and may therefore offer additional value, but it is still not breast cancer-specific [92,93]. By contrast, the Caprini score is primarily applicable to perioperative risk stratification [94]. For ease of clinical application, the indications, strengths, and major limitations of commonly used VTE risk assessment tools in BC are summarized in Table 3.

Accordingly, VTE risk assessment in BC should be performed throughout the disease course and repeated at key time points, including the early post-diagnosis period, disease progression, treatment transitions, hospitalization, and surgery. Particular vigilance is warranted in patients with aggressive subtypes, advanced disease, prior VTE, obesity, older age, central venous catheters, or a substantial comorbidity burden. In recent years, machine learning approaches have also been explored for VTE-related risk prediction. The study by Mora et al. suggests that such models may have potential for stratifying the risk of major bleeding during anticoagulant therapy [95]. However, because the available evidence is still derived largely from non-breast cancer-specific populations, these approaches should currently be regarded as complementary rather than as substitutes for existing clinical assessment tools.

### 6.2. Biomarkers and Clinically Available Indicators

Potential indicators of breast cancer-associated VTE can be broadly divided into mechanism-related biomarkers and clinically available indicators. Mechanism-related biomarkers, including TF, NETs, extracellular vesicles, and PAI-1, mainly contribute to understanding tumor-associated hypercoagulability and thrombo-inflammatory mechanisms; however, their use in routine clinical practice remains limited.The latter, such as D-dimer, platelet count, basic coagulation parameters, and hematologic indices derived from routine blood tests, are more accessible and better suited for repeated monitoring [96]. Current evidence suggests that the neutrophil-to-lymphocyte ratio (NLR), platelet-to-lymphocyte ratio (PLR), and systemic immune–inflammation index (SII) have prognostic value in patients with pulmonary embolism, with NLR showing relatively more stable prognostic performance [97]. However, the available evidence is derived primarily from general PE populations, and its value in BC remains to be further validated. At present, these indicators are therefore better regarded as complementary tools for the clinical evaluation of breast cancer-associated VTE.

### 6.3. Prevention Strategies

VTE prevention in patients with BC should be based on risk stratification rather than applied routinely. Current ASH, ASCO, and ESC guidelines do not support routine prophylactic anticoagulation for all ambulatory patients receiving systemic therapy but instead recommend considering pharmacologic thromboprophylaxis only in those assessed to be at high risk and without substantial bleeding risk or clinically significant drug–drug interactions. Specifically, ASH does not recommend routine prophylaxis in low-risk ambulatory patients, while direct oral anticoagulants (DOACs) or low-molecular-weight heparin (LMWH) may be considered in high-risk outpatients [88]. ASCO suggests that apixaban, rivaroxaban, or LMWH may be considered in high-risk patients [89]. ESC likewise emphasizes that primary prophylaxis in the ambulatory setting should be limited to high-risk patients without contraindications [87].

For hospitalized patients, the decision to initiate prophylaxis should still be based on a comprehensive assessment of reduced mobility, infection, tumor stage, recent surgery, and overall clinical status. In patients with active malignancy and no major contraindications, prophylaxis should generally be considered during hospitalization. In the perioperative setting, ASCO recommends that patients undergoing major cancer surgery generally receive postoperative pharmacologic prophylaxis for at least 7–10 days, and the 2023 update further included apixaban and rivaroxaban as options for extended postoperative prophylaxis [89]; however, the supporting evidence is derived mainly from pan-cancer surgical populations. ESC places greater emphasis on clinical scenarios such as hospitalization, reduced mobility, and the postoperative period, with perioperative prophylaxis representing a major focus [87]. In patients with BC, the overall risk of VTE in breast surgery is lower than that in major abdominopelvic surgery; therefore, extended prophylaxis strategies derived from broader oncology populations should not be applied mechanically. For most patients, management should be based on early ambulation, mechanical prophylaxis, and individualized risk assessment, with pharmacologic prophylaxis reserved for those with multiple high-risk features, such as older age, obesity, prior VTE, reconstructive surgery, or other major risk factors. Overall, VTE prevention in BC should be tailored to specific clinical settings, including the ambulatory, inpatient, and perioperative settings, rather than applied through a uniform strategy [98]. For ease of comparison of the different emphases of the ASH, ASCO, and ESC guidelines on risk assessment, prevention, and treatment management, their main recommendations are summarized in Table 4.

### 6.4. Diagnostic Strategies

Clinical manifestations of VTE in patients with BC are often nonspecific. DVT may present with limb swelling, pain, or erythema, whereas upper-extremity DVT should be distinguished from postoperative lymphedema and catheter-related complications [99]. PE commonly presents with dyspnea, chest pain, or rapid breathing, and in severe cases may lead to hemodynamic instability [4]. Because the malignancy itself and anticancer treatment may produce similar manifestations, diagnosis should be based on an integrated assessment of medical history, physical examination, laboratory findings, and imaging studies. D-dimer has limited specificity in patients with cancer, but it may still serve as an adjunctive indicator. Venous ultrasonography is the first-line test in suspected DVT [100], whereas computed tomography pulmonary angiography (CTPA) is the first-line imaging modality in suspected PE; ventilation–perfusion (V/Q) scanning is appropriate when contrast agents are contraindicated or in specific clinical settings [101]. In patients with BC, particular attention should also be paid to catheter-related upper-extremity thrombosis and incidental PE, the latter of which is generally managed as symptomatic VTE in patients with active malignancy.

### 6.5. Anticoagulant Therapy and Clinical Decision-Making

Patients with BC who develop VTE should receive anticoagulant therapy unless there is a clear contraindication. Current ASH, ASCO, and ESC guidelines generally support the use of LMWH or DOACs for the initial and short-term treatment of cancer-associated VTE, although their emphasis differs somewhat. ASH supports the use of either DOACs or LMWH in appropriate patients and tends to favor anticoagulation beyond 6 months in those with active cancer. The 2023 ASCO update further added apixaban as a treatment option for cancer-associated VTE. ESC places greater emphasis on individualized drug selection according to bleeding risk, drug–drug interactions, tumor site, and clinical stability. To improve clinical applicability, the main considerations for choosing between DOACs and LMWH in different clinical settings are summarized in Table 5.

**Figure 3:**
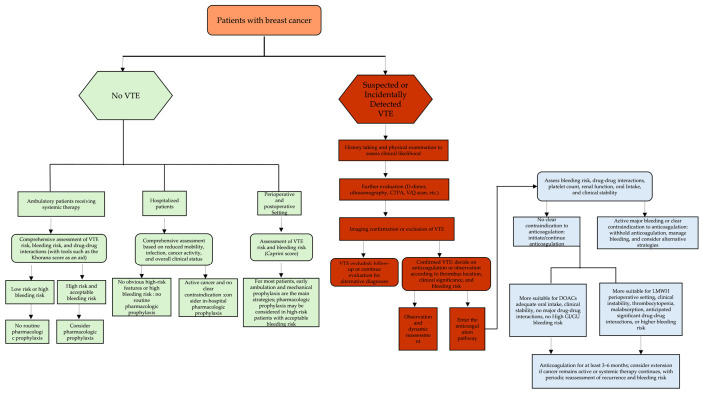
Overall clinical decision pathway for breast cancer-associated VTE. The pathway covers risk-stratified prevention, diagnostic evaluation of suspected or incidentally detected VTE, and individualized anticoagulant management according to bleeding risk, drug–drug interactions, platelet count, renal function, oral intake, and clinical stability. This figure was designed and created by the authors using Microsoft PowerPoint. Abbreviations: VTE, venous thromboembolism; CTPA, computed tomography pulmonary angiography; V/Q, ventilation–perfusion; DOACs, direct oral anticoagulants; LMWH, low-molecular-weight heparin.

In patients with BC, DOACs are generally more suitable for those who can take oral medication reliably, have good adherence, acceptable renal function, relatively stable platelet counts, and no major drug–drug interactions or high gastrointestinal/genitourinary bleeding risk. By contrast, LMWH is more appropriate in the perioperative setting, in clinically unstable patients, in those with nausea, vomiting, or malabsorption, in the presence of thrombocytopenia or a higher bleeding risk, or when significant drug–drug interactions are anticipated [102]. Because BC is not typically classified as a gastrointestinal or genitourinary high-bleeding-risk malignancy, DOACs are generally feasible in most clinically stable patients [103]. Nevertheless, particular attention should still be paid to their potential interactions with endocrine therapy, CDK4/6 inhibitors, and other anticancer agents. The duration of anticoagulation should generally be at least 3–6 months. If the tumor remains active or systemic anticancer therapy is ongoing, the decision to extend treatment should be based on a reassessment of the risks of recurrence and bleeding. Incidental VTE is generally managed as a symptomatic disease in patients with active malignancy. Thrombolysis, catheter-directed intervention, thrombectomy, and inferior vena cava filter placement should be reserved for special circumstances, such as high-risk pulmonary embolism, anticoagulation failure, or a clear contraindication to anticoagulation, and should not be used routinely [101]. Based on the above principles of risk assessment, prevention, diagnosis, and treatment, the overall clinical decision pathway for breast cancer-associated VTE is shown in Figure 3.

## 7. Future Perspectives on the Prevention and Management of Breast Cancer-Associated VTE

### 7.1. Screening and Validation of Biomarkers

Early identification of breast cancer-associated VTE still relies largely on clinical risk assessment, D-dimer testing, and imaging, as highly specific screening tools suitable for routine clinical use remain lacking [104]. In recent years, tissue factor (TF), extracellular vesicle-associated TF activity (EV-TF), neutrophil extracellular trap (NET)-related components, inflammatory cytokines, and markers of endothelial injury have shown promise [105]. However, the lack of standardized assay methods, sample processing procedures, and interpretation criteria limits comparability across studies and hinders clinical translation. Future studies should build on an improved understanding of the underlying molecular mechanisms to identify more practical and clinically accessible markers for early detection and risk stratification of VTE. At the same time, candidate biomarkers should undergo standardized validation, including external validation in prospective, multicenter cohorts, to further assess their added value beyond existing risk assessment tools.

### 7.2. Optimization of Risk Prediction Models

Most currently available risk assessment tools have been developed in general oncology populations, and their applicability to patients with BC remains limited. Future model optimization should place greater emphasis on BC specificity and improve the ability to capture differences in risk across molecular subtypes, treatment stages, and dynamic changes over time. In recent years, machine learning-based approaches have shown promise in predicting cancer-associated VTE and anticoagulation-related bleeding risk by integrating multiple clinical and laboratory variables. However, validation evidence for these models in BC cohorts remains insufficient, and at present they are better regarded as complementary tools rather than replacements for existing assessment methods. Future work should focus on developing breast cancer-specific prediction models and strengthening the external validation and refinement of existing models [106].

### 7.3. Therapeutic Perspectives on Tumor-Thrombosis Interactions

As understanding of the interplay among tumor-associated hypercoagulability, thrombo-inflammatory responses, and the host microenvironment in breast cancer-associated VTE continues to deepen, future therapeutic strategies may extend beyond conventional anticoagulation toward novel approaches targeting tumor–thrombosis interactions.

Among these, factor XI/XIa-targeted strategies have attracted attention because they may reduce bleeding events while preserving antithrombotic efficacy, although their efficacy and safety in cancer-associated VTE still require further investigation [107]. In addition to direct anticoagulant approaches, adjunctive therapies with pleiotropic effects have also attracted growing interest. Statins are a representative example. Current evidence suggests that statins may influence VTE risk and clinical outcomes by modulating inflammatory responses, improving endothelial function, and affecting the balance between coagulation and fibrinolysis [108]. Some studies have also suggested that statins may improve outcomes in patients with acute pulmonary embolism. However, these data are derived mainly from observational studies or non-breast cancer-specific populations, and their clinical value in breast cancer-associated VTE remains to be clarified.

### 7.4. Breast Cancer-Specific Evidence Gaps

Many current conclusions regarding breast cancer-associated VTE are still derived primarily from pan-cancer studies, and breast cancer-specific evidence remains limited. In particular, clinical data on the incidence, recurrence, and bleeding risk of VTE remain limited in patients with highly aggressive BC, pregnancy-associated BC, older age, renal insufficiency, or long-term exposure to endocrine therapy or CDK4/6 inhibitors. Future research should place greater emphasis on breast cancer-specific prospective cohorts and real-world studies to better define the true risk profiles across molecular subtypes, treatment exposures, and disease stages. Standardized outcome assessment incorporating VTE occurrence, recurrence, bleeding, treatment interruption, and long-term prognosis will be essential to improve the relevance and clinical applicability of the evidence.

## 8. Conclusions

Breast cancer-associated VTE is an important complication in the course of BC care and is closely linked to treatment interruption, poor prognosis, and an increased risk of death. Its risk is markedly time-dependent and varies across disease stages and treatment exposures, warranting sustained clinical attention. Current evidence indicates that, in addition to general factors such as age and comorbidity burden, the development of breast cancer-associated VTE is also closely related to tumor stage, molecular subtype, tumor burden, and treatment-related exposures, including surgery, systemic therapy, endocrine therapy, and central venous catheterization.

Mechanistically, breast cancer-associated VTE does not arise from a single factor, but rather from the combined effects of a tumor-associated procoagulant phenotype, inflammatory responses, platelet activation, endothelial injury, and alterations in the tumor microenvironment. Tissue factor, procoagulant extracellular vesicles, neutrophil extracellular traps, and related inflammatory pathways may collectively promote VTE by enhancing coagulation activation, inducing endothelial dysfunction, and amplifying thrombo-inflammatory responses. At the same time, current evidence suggests that coagulation activation and thrombosis-related changes may in turn affect tumor cell survival, invasion, and distant dissemination, indicating a bidirectional relationship between BC and thrombosis.

From a clinical perspective, the identification, prevention, and treatment of breast cancer-associated VTE should be based on dynamic risk stratification and a comprehensive assessment of patient-related factors, tumor biological characteristics, treatment exposures, and bleeding risk. Anticoagulation remains the cornerstone of management, but perioperative status, drug–drug interactions, bone marrow suppression, and thrombocytopenia may all influence treatment selection and safety. Management, therefore, requires a careful balance between antithrombotic benefit and bleeding risk. Rather than directly extrapolating evidence from pan-cancer populations, clinical decision-making in breast cancer-associated VTE should be individualized according to key time windows during the disease course and the specific treatment context.

Overall, advances in the study of breast cancer-associated VTE have not only deepened our understanding of its pathophysiology but have also provided an important basis for identifying high-risk patients, exploring biomarkers, and developing more precise prevention and treatment strategies. Future efforts should further strengthen breast cancer-specific prospective studies and real-world evidence to optimize risk prediction, early diagnosis, and individualized intervention and ultimately improve the overall prognosis of patients with BC.

## Figures and Tables

**Table 1:** Representative studies on the epidemiology and time-dependent risk of VTE in patients with breast cancer.

Study	Year	Population/Clinical Setting	Absolute Risk	Relative Risk/Comparison	Key Finding
Mulder et al. [7]	2021	Population-based cohort: patients with cancer vs. non-cancer controls	12-month cumulative incidence of VTE: 2.3% vs. 0.35%	Higher risk in patients with cancer	Indicates that cancer increases not only the relative risk of VTE but also the absolute thrombotic burden.
Walker et al. [8]	2016	Breast cancer patients during chemotherapy and the early post-treatment period	Annualized incidence is approximately 6%	Higher than in most other treatment phases	Chemotherapy and the first month after treatment represent a high-risk window for VTE.
Walker et al. [8]	2016	Breast cancer patients during tamoxifen treatment	Annualized incidence is approximately 2%	Approximately 4-fold higher than in the pretreatment period	VTE risk remains elevated during endocrine treatment exposure, particularly with tamoxifen.
Ohsumi et al. [9]	2023	Breast cancer subgroup of the Cancer-VTE Registry: baseline screening before treatment initiation and approximately 1 year of follow-up	Baseline VTE: 2.0%; composite VTE during follow-up: 0.5%; all-cause mortality: 2.1%	All-cause mortality exceeded composite VTE incidence	Provides recent breast cancer-specific prospective cohort data and suggests that death is a non-negligible competing event.
Londero et al. [10]	2022	Female patients undergoing breast surgery: postoperative follow-up	3 months: 0.4%; 1 year: 0.6%; 2 years: 0.9%; 5 years: 1.2%; 10 years: 1.7%	Cumulative risk increased over time	Provides long-term cumulative incidence data and highlights the time-dependent nature of postoperative VTE risk in breast surgery patients.

Abbreviation: VTE, venous thromboembolism.

**Table 2:** Comparison of VTE risk associated with endocrine therapies in breast cancer and potential explanations for discrepant findings.

Study	Design/Population	Exposure	Main VTE-Related Finding	Interpretation
Hernandez et al., 2009 [26]	Danish population-based cohort; early breast cancer	Tamoxifen	Increased DVT/PE risk during the first 2 years; RR 3.5, 95% CI 2.1–6.0	Tamoxifen is associated with increased early VTE risk
Walker et al., 2016 [8]	English population-based cohort; 13,202 breast cancer patients	Tamoxifen; aromatase inhibitors	Tamoxifen: HR 5.5, 95% CI 2.3–12.7 in the first 3 months; aromatase inhibitors: HR 0.8, 95% CI 0.5–1.4	Short-term VTE risk appears mainly related to tamoxifen.
Xu et al., 2019 [27]	Breast cancer survivors receiving long-term endocrine therapy	Aromatase inhibitors vs. tamoxifen	Aromatase inhibitors: HR 0.59, 95% CI 0.43–0.81 vs. tamoxifen	Aromatase inhibitors show lower VTE risk than tamoxifen
Blondon et al., 2022 [28]	Prospective HEMOBREAST cohort; localized breast cancer	Tamoxifen; aromatase inhibitors	Tamoxifen increased thrombin generation and reduced protein C pathway sensitivity; aromatase inhibitors did not show similar changes.	Biomarker evidence supports a greater procoagulant effect of tamoxifen.

Abbreviations: AI, aromatase inhibitor; VTE, venous thromboembolism.

**Table 3:** Applicability and limitations of common VTE risk assessment tools and emerging predictive methods in patients with breast cancer.

Model/Method	Main Clinical Setting	Main Strengths	Main Limitations in Breast Cancer
Khorana score	Initial risk screening in ambulatory patients receiving systemic therapy	Simple, widely used, and convenient for the rapid identification of high-risk ambulatory patients	Developed in a pan-cancer outpatient population; does not incorporate breast cancer-specific factors such as molecular subtype, endocrine therapy, CDK4/6 inhibitor exposure, or central venous catheterization and may therefore underestimate risk heterogeneity in some patients
COMPASS-CAT score	Risk stratification in ambulatory patients with solid tumors	Incorporates more information on treatment exposures and comorbidities; may provide additional value in ambulatory patients with solid tumors	Still a pan-cancer model; lacks a comprehensive assessment of breast cancer subtype, treatment characteristics, and relevant biomarkers, and is therefore not breast cancer-specific
Caprini score	Perioperative risk stratification	Useful for perioperative VTE risk stratification and may assist postoperative prophylaxis decisions.	Primarily applicable to surgical settings and difficult to extend to the chemotherapy period, endocrine therapy period, or long-term survivorship.
Machine learning methods/emerging predictive tools	Prediction of VTE risk or anticoagulation-related bleeding risk	May integrate multidimensional clinical and laboratory information and improve recognition of complex risk patterns	External validation remains limited; mature breast cancer-specific models are lacking; interpretability and clinical implementability are still suboptimal, so these tools cannot yet replace current clinical assessment methods

Abbreviations: VTE, venous thromboembolism; COMPASS-CAT, Clinical Prediction Score for Cancer-Associated Thrombosis; CDK4/6, cyclin-dependent kinase 4/6; AI, artificial intelligence.

**Table 4:** Comparison of the main recommendations of the ASH, ASCO, and ESC guidelines on risk assessment, prevention, and treatment of cancer-associated VTE.

Guideline/Document	Principles of Risk Assessment	Primary Thromboprophylaxis in Ambulatory Patients	Thromboprophylaxis in Hospitalized/Perioperative Patients	Key Treatment Recommendations
ASH 2021 guideline	Risk assessment should not rely solely on a single score but should integrate thrombotic risk, bleeding risk, and the overall clinical context.	Routine prophylaxis is not recommended for low-risk ambulatory patients; LMWH or DOACs may be considered in high-risk ambulatory patients.	Prophylaxis may be considered in appropriate hospitalized patients with cancer; pharmacologic prophylaxis may also be used in patients undergoing cancer surgery.	DOACs or LMWH may be used for initial treatment; in patients with active cancer, extended anticoagulation (>6 months) is generally favored.
ASCO 2020 guideline + 2023 update	Decisions should not be based solely on a single risk score but should incorporate thrombotic risk, bleeding risk, treatment exposures, drug–drug interactions, and overall clinical status.	Routine prophylaxis is not recommended for all ambulatory patients receiving systemic therapy; apixaban, rivaroxaban, or LMWH may be considered in high-risk patients.	Postoperative prophylaxis is a key focus; pharmacologic prophylaxis is generally recommended for at least 7–10 days after cancer surgery. The 2023 update added apixaban and rivaroxaban as options for extended postoperative prophylaxis.	DOACs or LMWH may be used for cancer-associated VTE; the 2023 update added apixaban as a treatment option. Incidental VTE is generally managed as a symptomatic disease.
ESC 2022 cardio-oncology guideline	Emphasizes dynamic assessment according to clinical setting, with individualized decision-making based on tumor characteristics, treatment-related factors, bleeding risk, drug–drug interactions, and patient preference.	Primary prophylaxis in the ambulatory setting should only be considered in high-risk patients without contraindications.	Greater emphasis is placed on hospitalization, reduced mobility, and the perioperative setting, which are regarded as major scenarios for considering prophylaxis.	Anticoagulant treatment should be individualized; incidental VTE is generally managed as symptomatic disease; anticoagulation is usually continued while cancer remains active.

Abbreviations: ASH, American Society of Hematology; ASCO, American Society of Clinical Oncology; ESC, European Society of Cardiology; VTE, venous thromboembolism; DOACs, direct oral anticoagulants; LMWH, low-molecular-weight heparin.

**Table 5:** Key considerations for choosing DOACs versus LMWH in the anticoagulant treatment of breast cancer-associated VTE.

Clinical Scenario	More Favorable for DOACs	More Favorable for LMWH
Oral intake/adherence	Able to take oral medication reliably; good adherence	Nausea, vomiting, malabsorption, or poor oral intake
Potential drug–drug interactions	No significant drug–drug interactions	Significant or anticipated drug–drug interactions
Bleeding risk	No obvious high gastrointestinal/genitourinary bleeding risk	Higher bleeding risk, especially when closer dose adjustment or temporary discontinuation may be needed
Platelet count/renal function	Relatively stable platelet count and acceptable renal function	Thrombocytopenia, or situations requiring greater flexibility in anticoagulant adjustment
Perioperative setting or clinical instability	Generally not the first choice	More appropriate
Overall candidate profile	Clinically stable patients with reliable oral intake and no major drug interactions	Patients in the perioperative setting, with clinical instability, thrombocytopenia, malabsorption, or other complex comorbid conditions

Abbreviations: DOACs, direct oral anticoagulants; LMWH, low-molecular-weight heparin; VTE, venous thromboembolism.

## Data Availability

Not applicable. This is a review article, and no original datasets or materials were generated or analyzed in this study.
